# Supplementary material for: Non-local X-ray intermolecular radiative decay probes solvation shell of ions in water
Source: Nat Commun. 2025 Nov 17;16:10046. doi: 10.1038/s41467-025-65581-7 (PMC12623427; doi:10.1038/s41467-025-65581-7)
Supplement: Supplementary file 1 — Supplementary Information [file 41467_2025_65581_MOESM1_ESM.pdf]

Supplementary Information for: Non-local X-ray intermolecular radiative decay probes solvation shell of ions in water

Johan Söderström[1*], Lucas M. Cornetta[2], Victor Ekholm[3], Vincenzo Carravetta[4], Arnaldo Naves de Brito[5], Ricardo Marinho[6], Marcus Agåker[1,3], Takashi Tokushima[3], Conny Såthe[3], Anirudha Ghosh[3], Dana Bloß[7], Andreas Hans[7], Florian Trinter[8], Iyas Ismail[9], Debora Vasconcelos[1], Joel Pinheiro[1], Yi-Ping Chang[10], Manuel Harder[10], Zhong Yin[11], Joseph Nordgren[1], Gunnar Öhrwall[3], Hans Ågren[1,12], Jan-Erik Rubensson[1], Olle Björneholm[1*]

[1]Department of Physics and Astronomy, Uppsala University, Uppsala, Sweden.

[2]Instituto de Física, Universidade de São Paulo, São Paulo, Brazil.

[3]MAX IV Laboratory, Lund University, Lund, Sweden.

[4]Institute of Chemical and Physical Processes, CNR-IPCF, Pisa, Italy.

[5]Institute of Physics Gleb Wataghin, State University of Campinas, Campinas, Brazil.

[6]Department of Physics, University of Brasilia, Brasilia, Brazil.

[7]Institute of Physics, University of Kassel, Kassel, Germany.

[8]Molecular Physics, Fritz-Haber-Institut der Max-Planck-Gesellschaft, 14195, Berlin, Germany.

[9]Sorbonne Université, CNRS, Laboratoire de Chimie Physique – Matière et Rayonnement, LCPMR, F-75005 Paris, France.

[10]European XFEL, 22689 Schenefeld, Germany.

[11]International Center for Synchrotron Radiation Innovation Smart, Tohoku University, 980-8577 Sendai, Japan.

[12]Faculty of Chemistry, Wroclaw University of Science and Technology, PL-50370 Wroclaw, Poland.

*Corresponding author(s). E-mail(s): Johan.Soderstrom@physics.uu.se;
Olle.Bjorneholm@physics.uu.se;
Contributing authors: lucas.m.cornetta@gmail.com;
victor.ekholm@maxiv.lu.se; vincenzo.carravetta@gmail.com;
arnaldo@ifi.unicamp.br; marinho.r@gmail.com;
marcus.agaker@physics.uu.se; takashi.tokushima@maxiv.lu.se;
conny.sathe@maxiv.lu.se; anirudha.ghosh@maxiv.lu.se;
dana.bloss@uni-kassel.de; hans@physik.uni-kassel.de;
trinter@fhi-berlin.mpg.de; iyas.ismail@sorbonne-universite.fr;
deboranb.fisica@gmail.com; joanfepi9@gmail.com; yi-ping.chang@xfel.eu;
manuel.harder@desy.de; zhong.yin.e2@tohoku.ac.jp;
joseph.nordgren@physics.uu.se; gunnar.ohrwall@maxiv.lu.se;
hans.agren@physics.uu.se; jan-erik.rubensson@physics.uu.se;

Supplementary Note 1 Additional experimental results

Supplementary Note 1.1 Experimental Results from P04 at PETRA III

For Mg^{2+} in water, preliminary experiments were also carried out at the P04 beamline of the synchrotron radiation facility PETRA III, DESY, Hamburg [1]. The results from these experiments are summarized in Supplementary Figure 1.

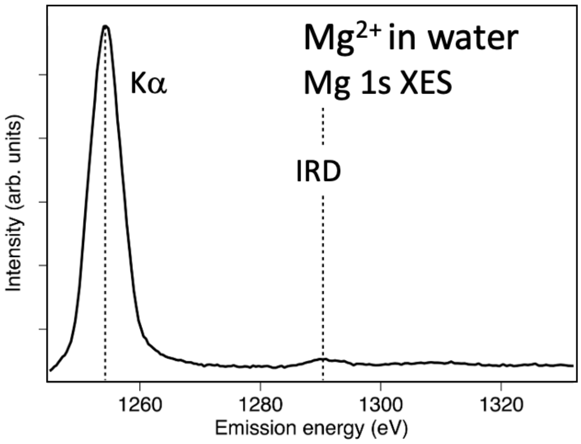

Supplementary Figure 1: The experimental X-ray spectrum after Mg^{2+} 1s ionization in the energy range of K_α and IRD measured at P04. The spectrum shown is a sum of several spectra recorded with different photon energies above and just below the Mg^{2+} 1s ionization threshold. Within the experimental resolution these spectra exhibit no significant differences. To improve the statistics, the spectra were therefore summed. The spectrum contains two peaks, of which the most intense one is the K_α, $Mg^{3+}\,1s^{-1} \rightarrow Mg^{3+}\,1s^2\,2p^{-1} + h\nu$. The weaker peak at 1290.4 eV agrees well with the energy estimated for the non-local radiative decay, $Mg^{3+}\,1s^{-1} + H_2O \rightarrow Mg^{2+} + H_2O^+\ \mathrm{val}^{-1} + h\nu'$. The vertical dashed lines are guides for the eye for the K_α and IRD features.

Supplementary Note 1.2 General applicability

IRD appears to be a general phenomena for ions in liquid surrounding. To highlight this we show preliminary data from both Cu^{2+} and F^- ions in aqueous solution in Supplementary Figure 2. Both of these spectra were measured under similar experimental conditions as both Na^+ and Mg^{2+} at the Veritas beamline, both with a concentration of 1 M.

In short the Cu^{2+} spectrum shows the elastic scattering at 0 eV energy loss, dd-excitations at 0.2 – 2 eV energy loss and IRD features involving water at 2 – 12 eV

energy loss. The latter energy coincides with what is typically labeled the charge-transfer band in cuprates [2]. Cu^{2+} ions was obtained by dissolving $CuCl_2$ in water, the photon energy was set to ~928.6 eV, corresponding to top of the L_3 resonance [3].

The emission spectrum from F^- shows the 2p → 1s emission at ~677 eV and a low energy feature. The low-energy feature is a part of the IRD features. Overlaid with the experimental spectrum is both the water valence UPS spectrum, as well as preliminary DFT calculations, these are both shifted to align with the peak at ~671.5 eV. F^- ions were obtained by dissolving KF in water, and the photon energy was set to ~689 eV, ~0.2 eV above the $1s^{-1}$ ionization energy.

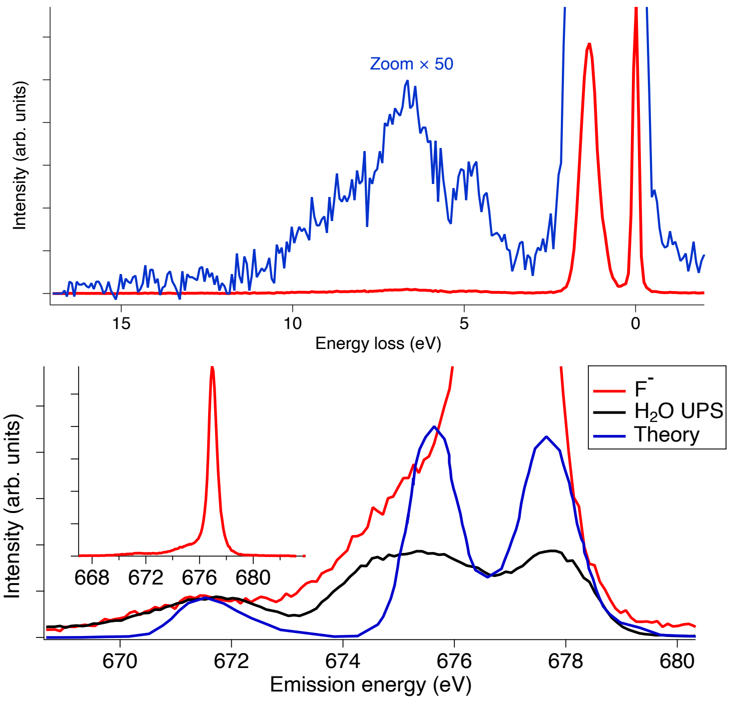

Supplementary Figure 2: Top panel: IRD signal from Cu^{2+} in water, see text for details. The blue curve is a 50× magnification of the red spectrum to highlight the IRD features. Bottom panel: IRD signal from F^- in water, see text for details. The spectrum shown has a vertical zoom of 10×, while the entire spectrum is shown in the inset. Overlaid with the experimental spectrum are preliminary DFT calculations as well as the water UPS spectrum, both aligned by eye to the peak at ~671.5 eV.

Supplementary Note 2 Additional Computational Details

Supplementary Note 2.1 Model System Structures

A number of model systems, namely clusters including a single cation surrounded by a variable number of water molecules, have been considered. The computational methods we employed varied from accurate ab-initio correlated methods for small models, to DFT and HF methods for the larger clusters, with the purpose to investigate different aspects of the IRD process and the formation of the corresponding bands.

Supplementary Figure 3 shows schematic illustrations of the used structures: symmetric D_{2h} $M^q[H_2O]_6$ structure (top), representative distorted $M^q(H_2O)_6$ structure (middle), and representative distorted $M^q(H_2O)_5Cl^-$ structure (bottom). The two representative, distorted, structures are average structures obtained from the MD trajectories by the GROMACS tool cluster.

Supplementary Note 2.2 Mulliken Charges

The Mulliken charges for the different electronic states of Na and Mg for the symmetric D_{2h} structure model are shown in Supplementary Table 1, see also Fig. 3 in the main text. We emphasize that while Mulliken charges provide insight into charge redistribution across ionization and subsequent electronic decay, they are not used to derive spectral intensities, which are governed by transition dipole matrix elements.

Supplementary Table 1: Mulliken charges for the electronic states (ground state, intermediate core-ionized state, as well as inner-valence (IV) and outer-valence (OV) one-hole final states, per atomic center, for both $M[H_2O]_6$, M=Na, Mg systems. Each of the oxygen lines represents a symmetric pair of oxygen atoms, whilst each of the hydrogen lines represents four hydrogen atoms. The bottom line, Water charge, represents the total charge on the water molecules. Data based on Open Shell-Restricted HF optimization of each specified state.

		Na							Mg						
States		ground	intermediate	final					ground	intermediate	final				
			1s hole	2p hole	IV hole	OV hole				1s hole	2p hole	IV hole	OV hole		
Quant.	Atom		$M1s^{-1}$	$M2p^{-1}$	$2a_1^{-1}$	$1b_2^{-1}$	$3a_1^{-1}$	$1b_1^{-1}$		$M1s^{-1}$	$M2p^{-1}$	$2a_1^{-1}$	$1b_2^{-1}$	$3a_1^{-1}$	$1b_1^{-1}$
1	M	0.95	1.48	1.53	1.20	0.87	0.87	0.86	1.35	1.75	2.20	1.22	1.21	1.25	1.21
2	O(1)	-0.42	-0.46	-0.48	-0.48	-0.16	-0.48	-0.48	-0.49	-0.47	-0.53	-0.53	-0.20	-0.53	-0.53
2	O(2)	-0.42	-0.46	-0.39	-0.26	-0.51	-0.08	-0.50	-0.49	-0.47	-0.56	-0.17	-0.56	-0.16	-0.55
2	O(3)	-0.42	-0.46	-0.50	-0.50	-0.50	-0.49	-0.02	-0.49	-0.47	-0.56	-0.56	-0.56	-0.54	-0.08
4	H(1)	0.21	0.27	0.25	0.25	0.34	0.25	0.25	0.30	0.34	0.33	0.33	0.41	0.33	0.33
4	H(2)	0.21	0.27	0.29	0.31	0.27	0.29	0.27	0.30	0.34	0.35	0.40	0.35	0.37	0.35
4	H(3)	0.21	0.27	0.26	0.26	0.26	0.26	0.27	0.30	0.34	0.34	0.34	0.34	0.35	0.35
Water charge		0.05	0.52	0.47	0.80	1.13	1.13	1.14	0.66	1.25	0.80	1.78	1.79	1.75	1.79

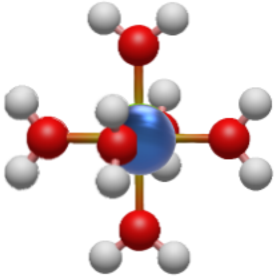

Symmetric D_{2h} $M^q(H_2O)_6$ structure

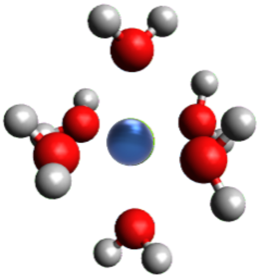

Representative distorted $M^q(H_2O)_6$ structure

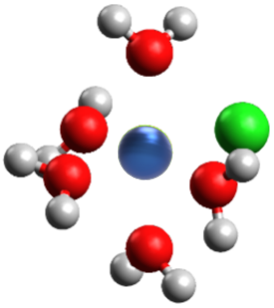

Representative distorted $M^q(H_2O)_5Cl^-$ structure

Supplementary Figure 3: Structures of the model systems. Top panel: The D_{2h} symmetric $M^q[H_2O]_6$ structure, consisting of a metal cation M^q (blue) surrounded by six water molecules in a symmetric D_{2h} structure, where the metal cation M^q is located at the origin, and with two water molecules in each Cartesian direction. Middle panel: The disordered $M^q[H_2O]_6$ structure obtained from Molecular Dynamics, used as a representative structure for the dynamically disordered structure in the solution. Bottom panel: The structure of $M^q[H_2O]_5Cl^-$, obtained by replacing one water molecule with a chloride ion (green), used as a representative structure for the case of ion pairing.

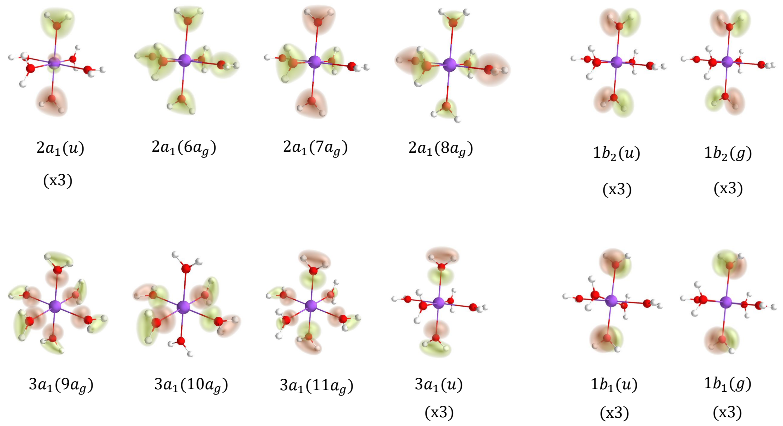

Supplementary Figure 4: Hybrid orbitals for the symmetric $M^q[H_2O]_6$ model system.

Supplementary Note 2.3 IRD and the One Center Approximation

X-rays are emitted as valence electrons fill atom-specific local core holes. This makes X-ray emission spectroscopy (XES) sensitive to the local electronic structure, and a merit of the method is that simplifying interpretation schemes can often be applied. Such schemes are typically based on the one-center model and the dipole selection rule [4]. Here the XES intensity depends on the local angular-momentum character of the core hole and the valence states, e.g., a quasi-atomic *1s* hole is filled by electrons from valence states of local p character only. The one-center approximation is commonly assumed to be valid, implying that XES reflects the local partial density of states.

In order to have a better view of the different mechanisms, we consider a simple molecular orbital approach. As described in the main manuscript, the molecular orbitals can then be written as $\psi_\mu = \sum_\nu C_\mu^\nu \chi_\nu$, where C_μ^ν is the hybrid orbital coefficient of atomic orbitals χ_ν. centered on the metal ion, as well as the atoms in the water molecules. Using a closed-shell HF reference for the $M^q[H_2O]_6$ model clusters, where $M^q = Na^+$, Mg^{2+} denotes the metal cation, the first-order X-ray emission amplitude from a particular MO (indexed by μ) to the ion $1s$ core hole is given by the dipole moment matrix element given by

$$\mathbf{T}_\mu = \langle \psi_{\mathrm{M}1s} | \mathbf{r} | \psi_\mu \rangle = \langle \psi_{\mathrm{M}1s} | \mathbf{r} | \psi_\mu^{\mathrm{M}} + \psi_\mu^{\mathrm{W}} \rangle = \mathbf{T}_\mu^{\mathrm{OC}} + \mathbf{T}_\mu^{\mathrm{MC}}, \quad (1)$$

where the sum $\psi_\mu^{\mathrm{M}} + \psi_\mu^{\mathrm{W}}$ explicitly decomposes the MO according to contributions arising from the metal- (M) and the surrounding water molecules- (W) centered basis functions. This allows us to conceptually decompose $\mathbf{T}_\mu$ into a one-center (OC) part – $\mathbf{T}_\mu^{\mathrm{OC}}$, due to transitions from M-centered wavefunctions, and a multi-center (MC) part – $\mathbf{T}_\mu^{\mathrm{MC}}$, due to transitions from W-centered wave functions. Since the intensity

is proportional to $|\mathbf{T}_\mu|^2$, one can write

$$f_\mu^{\mathrm{IRD}} = f_\mu^{\mathrm{OC}} + f_\mu^{\mathrm{MC}} + f_\mu^{\mathrm{I}} \propto |\mathbf{T}_\mu^{\mathrm{OC}}|^2 + |\mathbf{T}_\mu^{\mathrm{MC}}|^2 + \left(\mathbf{T}_\mu^{\mathrm{OC}} \cdot \mathbf{T}_\mu^{\mathrm{MC}} + c.c.\right) = |\mathbf{T}_\mu|^2, \quad (2)$$

where f_i^{I} is an interference term, and c.c. is the complex conjugate term. Two approximations within the MO picture have been considered for the emission spectra. The first one is based on the evaluation of $|\mathbf{T}_\mu|^2$ itself, called MO→MO calculation. The second is the so-called one-center approximation (OCA), in which we approximate $|\mathbf{T}_\mu|^2 \sim |\mathbf{T}_\mu^{\mathrm{OC}}|^2$, i.e. neglecting all MC contributions. A comparison between these two approaches provides information on the magnitude of each mechanism to the IRD intensity.

Supplementary Table 2 summarizes the MO→MO and the OCA calculations for the small model systems, showing individual contributions from atomic orbitals (AO) of the basis set and their respective MO coefficients. Our idealized molecular models for $Na^+[H_2O]_6$ and $Mg^{2+}[H_2O]_6$ were investigated, with the purpose of better understanding the competitive mechanisms within the IRD processes. The choice of the number of molecules used in these cluster models was $n = 6$ for both systems, consistent with the first solvation shells in aqueous solutions. For simplicity, a symmetric D_{2h} structure has been considered, where the metal cation is located at the origin and with two water molecules disposed along each cartesian direction. The resulting valence MO's are shown in Supplementary Figure 4 according to the different symmetries. The symmetry-adapted aug-cc-pVTZ basis set has been used, and we have labeled each equivalent pair of oxygen atoms as O(1), O(2), and O(3). The distances R between the metal ion and the oxygen atoms are the same in all three directions and $R = 2.3$ Å and $R = 2.1$ Å have been used for Na^+ and Mg^{2+}, respectively, also motivated by the behavior of the first solvation shells.

The *gerade* MO's have zero transition moments, thus we only discuss the *ungerade*(u) orbitals. Each symmetry-adapted $2a_1(u)$, $1b_2(u)$, $3a_1(u)$ and $1b_1(u)$ term denotes a set of three orbitals at once, associated with the b_{1u}, b_{2u} and b_{3u} components, in which the MO coefficients are the same, although interchangeable between O(1), O(2) and O(3) centers. AO's that have zero $\langle \mathrm{M}1s|r_i|\chi_\nu\rangle$ elements are not shown, nor are the contributions from $2p$ orbitals on the hydrogen atoms. Furthermore, the OCA calculations take the $np \to 1s$ atomic contributions for all np, $n = 2, 3, 4, 5$ on the metal ion into account, but only the most significant $n = 2$ and $n = 3$ contributions are shown in Supplementary Table 2 for simplicity.

Comparing the values for $|\mathbf{T}_\mu^{\mathrm{OC}}|^2$, representing OCA taking only contributions from metal ion orbitals, $|\mathbf{T}_\mu^{\mathrm{MC}}|^2$, representing cross-transitions from oxygen orbitals, and $|\mathbf{T}_\mu|^2$, representing MO → MO, which includes contributions from both metal ion and oxygen orbitals, we see that the $|\mathbf{T}_\mu^{\mathrm{MC}}|^2$ values are much smaller than $|\mathbf{T}_\mu^{\mathrm{OC}}|^2$. This is illustrated in Supplementary Figure 6, which shows that the spectra simulated using OCA and MO → MO are very similar in shape and intensity. The result from a Restricted Active Space Self-Consistent Field (RASSCF) calculation is also included, which agrees rather well with OCA and MO → MO regarding the spectral shape, but differs in IRD/K_α intensity ratio. This implies that the cross-transitions only weakly affect the total intensity, and that OCA describes the intensity quite well.

Supplementary Table 2: Summary of the most relevant transition-related quantities within the Molecular Orbital perspective obtained for the symmetric model systems $M^q[H_2O]_6$, where M^q=Na^+ (considering R = 2.3 Å) and Mg^{2+} (considering R = 2.1 Å). The quantities correspond to the following. Rows 5-14: Symmetry allowed cartesian components of the transition dipole moments $\mathbf{T}_\mu = \langle \psi_{\mathrm{M}1s} | \mathbf{r} | \psi_\mu \rangle$, in atomic units, resolved in the symmetry adapted atomic-orbitals χ_ν used to resolve the molecular orbitals $\psi_\mu = \sum_\nu C^\nu_\mu \chi_\nu$. Note that only contributions from $2p$ and $3p$ orbitals are listed, contributions from orbitals with n≥4 are included in the the sums of rows 15-18. Column 1 lists the involved atomic orbitals χ_ν, columns 2 and 7 present the atomic transition dipole moments $\mathbf{t} = \langle \mathrm{M}1s | r_i | \chi_\nu \rangle$ and, finally, the sets of columns 3-6 and 8-11 show the MO coefficients C^ν_μ of for inner-valence (IV) and outer-valence (OV) orbitals. Row 15: $|\mathbf{T}^{\mathrm{OC}}_\mu|^2$, the one-center contributions to the transition dipole moments. Row 16: $|\mathbf{T}^{\mathrm{MC}}_\mu|^2$, the multi-center contributions to the transition dipole moments. Row 17: $|\mathbf{T}_\mu|^2$ the total transition dipole moments. Row 18: Emission energies obtained from the RASSCF calculation (see text). Remaining data based on Open Shell-Restricted HF optimization of each specified state.

	Na					Mg				
		C^ν_μ					C^ν_μ			
		IV	OV				IV	OV		
χ_ν	t ($\times 10^{-3}$)	$2a_1(u)$	$1b_2(u)$	$3a_1(u)$	$1b_1(u)$	t ($\times 10^{-3}$)	$2a_1(u)$	$1b_2(u)$	$3a_1(u)$	$1b_1(u)$
$\mathrm{M}2p_i$	49.11	-0.27	0.01	0.08	0.01	47.05	-0.02	-0.01	0.03	0.02
$\mathrm{M}3p_i$	-4.53	-0.12	-0.03	-0.08	0.18	-6.78	0.10	-0.18	-0.35	0.12
$\mathrm{M}4p_i$	0.45	0.05	0.01	0.11	-0.10	0.93	-0.12	0.18	0.18	-0.15
$\mathrm{O}(1)2s$	0	0.84	0	-0.28	-0.05	0	0.86	0	-0.23	-0.07
$\mathrm{O}(1)2p_i$	0.04	0	-0.74	0.04	-0.01	0.06	0	-0.74	0.11	-0.01
$\mathrm{O}(2)2p_i$	0.04	0.11	0.04	0.81	0.09	0.06	0.10	0.09	0.79	0.14
$\mathrm{O}(3)2p_i$	0.04	0	-0.01	-0.08	0.91	0.06	0	-0.02	-0.12	0.91
$\mathrm{O}(1)3p_i$	0.00	0	0.02	0.01	0	0.01	0	0.02	0	0
$\mathrm{O}(2)3p_i$	0.00	0.01	0	-0.01	0	0.01	0	0	-0.01	0
$\mathrm{O}(3)3p_i$	0.00	0	0	0	0	0.01	0	0	0	0
$\lvert\mathbf{T}^{\mathrm{OC}}_\mu\rvert^2(\times 10^{-6})$		152.00	0.51	19.73	0.11		2.89	0.98	16.20	0.03
$\lvert\mathbf{T}^{\mathrm{MC}}_\mu\rvert^2(\times 10^{-6})$		0.01	0	0.07	0		0.02	0.01	0.03	0
$\lvert\mathbf{T}_\mu\rvert^2(\times 10^{-6})$		150.06	0.48	17.39	0.12		2.41	0.80	14.74	0.01
Emission energy (eV)		1044.9	1058.8	1062.9	1065.2		1278.7	1293.6	1295.8	1299.9

Supplementary Note 2.4 Dynamically Disordered Structural Model

The calculations presented in this section were performed for $Na^+[H_2O]_6$ and $Mg^{2+}[H_2O]_6$ representative clusters derived from molecular dynamics calculations. These indicate that the most typical configuration for the first solvation shell is made up of 6 water molecules for both cations, see the middle panel of Supplementary Figure

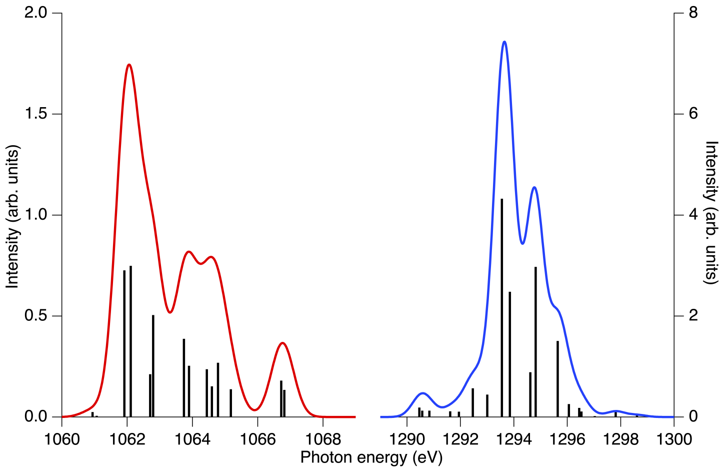

Supplementary Figure 5: XES-HF spectrum of the average clusters $Na^{+}[H_2O]_6$ (left) and $Mg^{2+}[H_2O]_6$ (right).

3. The main difference is the average distances between the cation and oxygen atoms of water, 2.4 Å for Na and about 2.0 Å for Mg, as derived from our MD simulations.

The independent particle approximation was employed to describe by a single determinant both the initial M$1s^{-1}$ relaxed core hole state of the X-ray emission process, and the final states with a frozen hole in the inner-valence or valence orbitals of $M^q[H_2O]_6^+$ clusters. The core hole state is better described by including the large effect of electron relaxation, This is accomplished by optimizing the orbitals by the usual self consistent procedure with the constraint of single occupancy of the core orbital. For the valence holes the frozen orbital approximation is, instead, generally assumed a better choice, considering that valence electron relaxation and valence electron correlation energies are often comparable in size and have opposite sign. The energies of the XES spectrum were obtained as the difference between the relaxed M$1s^{-1}$ core energy and the frozen valence energies, while the spectral intensities for the spontaneous emission process were obtained from the transition moment computed between the two determinants, in the dipole approximation. With the complete basis set of the $M^q[H_2O]_6^+$ cluster, the ground state of the single M^q cation was also obtained, in order to evaluate the overlap between occupied orbitals of the M^q cation and the occupied orbitals of the cluster.

The computed XES spectra, limited to the high energy emission, already presented in Fig. 2 of the main paper, are shown in Supplementary Figure 5 in more detail. Original theoretical data are represented by black bars (intensity in arbitrary units), while the red or blue solid lines show their convolution with a Gaussian (FWHM=0.8 eV) to facilitate the comparison with the experimental spectra.

An analysis of the origin of the intensity of the main XES band and the IRD bands in the spectrum of the $Na^{+}[H_2O]_6$ and $Mg^{2+}[H_2O]_6$ cluster derived from the MD, can be performed using the data reported in Supplementary Tables 3 and 4, respectively.

Na			Occupied in free ion			Unoccupied in free ion				Main source of electron
	t_{Nanl}		4.9×10^{-2}			0	-0.45×10^{-2}			
i	E(ev)	Int.	2px	2py	2pz	3s	3px	3py	3pz	
9	1005.54	34.98	0.0029	0.0018	0.0000	0.0001	0.0000	0.0000	0.0000	Na 2s
10	1040.17	111.08	0.6251	31.9217	48.2016	0.1653	2.3422	0.0157	0.4290	Na 2p
11	1040.18	126.92	91.3305	0.3948	0.1176	0.2823	0.0045	0.7066	0.5833	
12	1040.23	127.92	0.0016	52.8804	39.7470	0.4699	0.0692	0.5102	0.3740	
13	1040.60	25.48	1.5698	13.3978	2.1968	5.1544	3.9905	1.9824	1.1546	
14	1040.71	19.28	5.1063	0.7134	7.0168	2.3414	2.4352	5.5708	3.2924	Water inner valence
15	1041.09	1.55	0.4001	0.2310	0.3417	3.1137	2.0871	0.1462	6.6134	
16	1041.33	5.68	0.8851	0.3962	2.1875	3.1351	5.4447	0.1375	7.1068	
17	1043.38	0.50	0.0626	0.0244	0.1544	4.0484	1.9921	11.1222	0.0991	
26	1061.91	0.72	0.0001	0.0072	0.0003	3.6641	0.0044	0.0425	0.1518	Water outer valence
27	1062.11	0.74	0.0005	0.0003	0.0109	1.0658	0.5840	0.0921	0.0206	
28	1062.70	0.21	0.0007	0.0007	0.0013	0.6003	0.1864	0.6814	0.0275	
29	1062.79	0.50	0.0047	0.0014	0.0030	0.3864	0.0716	0.1486	0.0069	
30	1063.73	0.38	0.0014	0.0007	0.0004	1.1598	0.0836	0.4895	1.0501	
31	1063.89	0.25	0.0006	0.0012	0.0011	0.4570	0.0057	0.0243	0.6185	
32	1064.44	0.23	0.0002	0.0005	0.0016	0.2090	0.0002	0.2868	0.0037	
33	1064.59	0.15	0.0017	0.0000	0.0005	0.5516	0.1596	0.4970	0.0015	
34	1064.78	0.26	0.0010	0.0009	0.0015	1.2461	0.0558	0.7209	0.2650	
35	1065.17	0.13	0.0002	0.0000	0.0009	0.0303	0.0884	0.0587	0.0968	
36	1066.71	0.18	0.0005	0.0002	0.0010	0.7013	0.3459	0.1802	0.0277	
37	1066.81	0.13	0.0000	0.0002	0.0002	0.3022	0.6346	0.1482	0.0141	

Supplementary Table 3: Contributions to the XES spectrum calculated for the average cluster $Na^+[H_2O]_6$ cluster. Column 1: index i of the occupied orbital involved in the decay; column 2: transition energy (eV); column 3: transition intensity (arbitrary units); columns 4, 5 and 6 : (percentage) overlap between the i:th orbital of the cluster and the three 2p orbitals occupied in the isolated cation; columns 7, 8, 9 and 10: overlap with the virtual orbitals *3s* and *3p* in the isolated cation; column 11: labels that identify the origin of the bands by grouping the single contributions. Row 2 shows the atomic transition moments t_{Nanl} between the core orbital and the occupied or unoccupied (n=2,3 ; l=s,p) valence orbitals of the cation. Data based on molecular orbitals optimized for the ground state by HF.

For both Na and Mg, the atomic bands due to decay from 2s and 2p are predicted to be energetically well isolated. Note that the decay from 2s, which is dipole forbidden in the free ion, becomes allowed due to the deformation of the spherical symmetry in the presence of water molecules. At higher emission energy than these two atomic bands is a weak, relatively narrow and well-resolved band, due to decay from cluster orbitals deriving from the $2a_1$ inner-valence orbitals of the water molecules. Finally, at even higher emission energy there is a structured larger band due to decay from cluster orbitals that are combinations of the $3a_1/1b_2/1b_1$ outer valence orbitals of water molecules. The percentage overlaps with the occupied *2p* orbitals of Na^+ and Mg^{2+} clearly show how the most appreciable IRD intensities correspond to appreciable overlap values, i.e. to a hybridization mechanism that mixes the orbitals of the cation

Mg			Occupied in free ion			Unoccupied in free ion				Main source of electron
	$t_{\mathrm{Mg}nl}$		4.7×10^{-2}			0	-0.75×10^{-2}			
i	E(eV)	Int.	2px	2py	2pz	3s	3px	3py	3pz	
10	1211.03	4.09	0.0002	0.0002	0.0001	0.0011	0.0000	0.0000	0.0000	Mg 2s
11	1251.19	213.75	9.8448	0.1161	90.0015	0.0000	0.0034	0.0023	0.0013	}
12	1251.22	213.97	26.9904	70.9727	2.0046	0.0003	0.0000	0.0017	0.0040	} Mg 2p
13	1251.25	214.11	63.1319	28.8831	7.9577	0.0000	0.0019	0.0011	0.0010	}
14	1272.93	0.20	0.0006	0.0007	0.0064	19.6584	4.4457	2.3805	0.4977	}
15	1273.09	0.48	0.0125	0.0035	0.0032	0.5195	2.0442	0.4152	21.4137	}
16	1273.29	0.26	0.0002	0.0010	0.0091	1.9074	5.1259	8.1070	0.4751	} Water
17	1274.04	0.17	0.0015	0.0023	0.0014	4.5710	1.8404	5.8810	6.8753	} inner
18	1274.41	0.16	0.0017	0.0030	0.0005	2.0998	7.2954	5.6789	0.2516	} valence
19	1275.49	0.35	0.0025	0.0057	0.0009	4.7862	7.5683	6.6547	0.1418	}
22	1290.46	0.18	0.0000	0.0004	0.0000	0.0125	0.7614	1.5813	0.9184	}
23	1290.57	0.12	0.0000	0.0000	0.0003	0.0654	1.3636	0.0673	0.0443	}
24	1290.83	0.12	0.0000	0.0000	0.0003	0.2281	1.7801	0.6706	0.0000	}
25	1291.61	0.11	0.0001	0.0000	0.0002	0.6950	0.3078	0.1568	1.7463	}
26	1291.94	0.10	0.0002	0.0000	0.0000	0.3661	0.1404	1.1950	0.3565	}
27	1292.46	0.56	0.0005	0.0004	0.0010	15.3684	0.2321	0.5804	0.0589	}
28	1293.00	0.44	0.0000	0.0002	0.0008	0.7411	0.7571	1.2746	0.2765	} Water
29	1293.55	4.32	0.0074	0.0011	0.0039	0.0537	0.0366	0.0070	1.4721	} outer
30	1293.84	2.48	0.0007	0.0020	0.0043	0.2403	0.0236	0.2547	0.0638	} valence
31	1294.61	0.88	0.0003	0.0004	0.0009	0.8138	0.0147	0.0456	0.3782	}
32	1294.81	2.97	0.0014	0.0031	0.0009	0.0166	1.0001	0.5138	0.3829	}
33	1295.64	1.50	0.0009	0.0015	0.0002	0.9406	0.2858	0.4912	0.0166	}
34	1296.06	0.25	0.0001	0.0006	0.0000	0.0000	0.1656	0.7476	0.0059	}
35	1296.44	0.18	0.0002	0.0002	0.0000	0.0158	0.5697	0.1356	0.3449	}
36	1296.53	0.10	0.0000	0.0000	0.0003	0.0000	0.0812	0.0283	0.9153	}

Supplementary Table 4: Contributions to the XES spectrum calculated for the average cluster $Mg^{2+}[H_2O]_6$. Column 1: index i of the occupied orbital involved in the decay; column 2: transition energy (eV); column 3: transition intensity (arbitrary units); columns 4, 5 and 6 : (percentage) overlap between the i:th orbital of the cluster and the three 2p orbitals occupied in the isolated cation; columns 7, 8, 9 and 10: overlap with the virtual orbitals *3s* and *3p* in the isolated cation; column 11: labels that identify the origin of the bands by grouping the single contributions. Row 2 shows the atomic transition moments $t_{\mathrm{Mg}nl}$ between the core orbital and the occupied or unoccupied (n=2,3 ; l=s,p) valence orbitals of the cation. Data based on molecular orbitals optimized for the ground state by HF.

with those of the solvent molecules. Examples of this are orbitals 27, 29 and 30 for Na, and 29, 30 and 32 for Mg. Cluster orbitals which are less affected by hybridization with the metal *2p* orbitals, but instead exhibiting substantial charge transfer from the water orbitals to the virtual *3s* and *3p* cation orbitals, correspond to lower intensities. Examples of this are orbitals 34 and 36 for Na, and 14 and 27 for Mg,

Supplementary Note 2.5 IRD and Distance Dependence

The efficiency of other non-local decay processes is known to decrease rapidly with increasing distance, for e.g., ICD, the efficiency is considered to scale with $1/R^6$ for

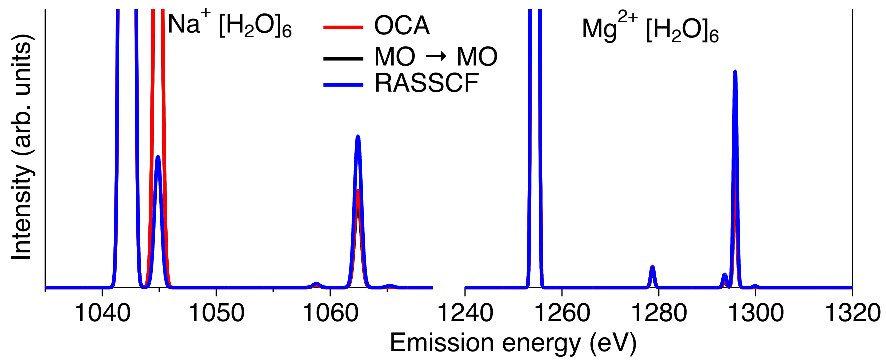

Supplementary Figure 6: Comparison between calculated XES spectra after M *1s* ionization for $Na^+[H_2O]_6$ and $Mg^{2+}[H_2O]_6$ obtained using OCA (red), MO → MO (black) and RASSCF (blue). Note that red and black curves overlap to a large extent.

longer distances [5]. The ability of IRD to probe the first solvation shell depends on the IRD efficiency decreasing rapidly with distance. We start by investigating the efficiency of IRD as a function of distance between the metal ion and the solvation shell water molecules of the idealized structures by examining spectra computed using Hartree Fock Restricted Active Space Self-Consistent Field (RASSCF) methodology (HF/RAS), shown in Supplementary Figure 7, for Na^+ and Mg^{2+} surrounded by one, four, and six water molecules in symmetric positions. The molecular orbitals were optimized for the ground, core-hole and final states.

From the calculations, the IRD/K_α intensity ratio was derived, see Supplementary Figure 8. As can be seen, the IRD/K_α intensity ratio increases with increasing number of water molecules, consistent with the IRD being caused by the solvation-shell water, and with decreasing distance.

A fit of the distance dependence of the IRD/K_α intensity ratio for n=1 to a $1/R^x$ function yields x≈6.4 for Na and x≈4.5 for Mg. The difference between the two ions can be attributed to the different amounts of hybridization that occur as a function of distance and orbital size. As the number of water molecules in a shell of radius R around the metal ion scales with $\sim R^2$, the values obtained for the $1/R^x$ distance dependence of the IRD/K_α intensity ratio imply that the first solvation shell will dominate the IRD process.

The dependence of the IRD/K_α intensity ratio on the number of neighboring water molecules opens the possibility of quantitatively analyzing the composition of the solvation shell, for example, in connection to ion pairing as discussed further below. For the experimentally determined M–O distances in solution, the calculated IRD/K_α ratio for n=6 is ∼1.0 % and ∼1.8 % for Na and Mg, respectively, to be compared to our experimental values of ∼1.0 % and ∼1.5 %. For Na the agreement is excellent, and for Mg an even better agreement was obtained with a more realistic structure model derived from MD simulations of the aqueous solution, as discussed in the main text.

Parallel to the increase of the IRD/K_α intensity ratio with decreasing distance, the lower panels of Supplementary Figure 8 shows how the energy splitting between

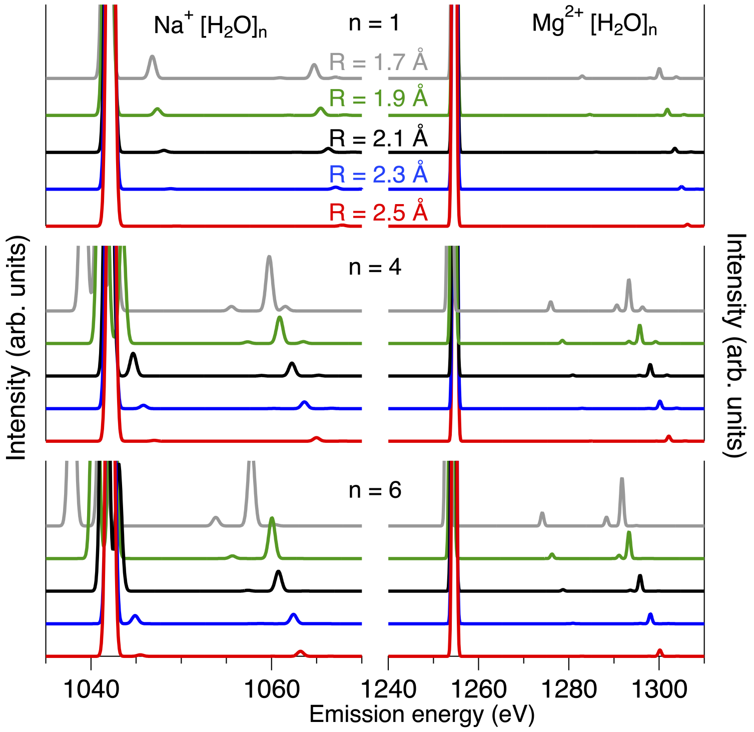

Supplementary Figure 7: Calculated XES spectra after M 1s ionization for $Na^{+}[H_2O]_n$ and $Mg^{2+}[H_2O]_n$ as function of M-water distance (R), and number of water molecules (n).

the K_α and IRD spectral features, the latter here defined as the energy of the main $3a_1$ peak, decreases with decreasing distance. This change can qualitatively be understood as being due to an increasing hybridization between orbitals on the ion and the water molecules with decreasing distance R. For the experimentally determined M–O distances in solution, the energy splitting obtained from calculation for n=6 is ∼21 eV for Na and ∼39 eV for Mg, respectively, in fair agreement with our experimental values of ∼23 eV for Na and ∼41 eV for Mg.

We conclude that both the IRD/K_α intensity ratio and energy splitting are strongly sensitive to distance and number of nearest neighbors, pointing towards using IRD as a probe to explore the solvation shell.

Supplementary Note 2.6 IRD and Solvation Shell (Dis)Order

In addition to energy shifts of the one-hole states, the spectra in Fig. 4 of the main paper also exhibit differences in relative intensity between the $1b_1$, $3a_1$, and $1b_2$ peaks. For both Na and Mg $1b_2$ is much weaker than the other two. The $1b_1$ and $3a_1$ features, however, exhibit differences between Na and Mg. For Na, they have roughly equal

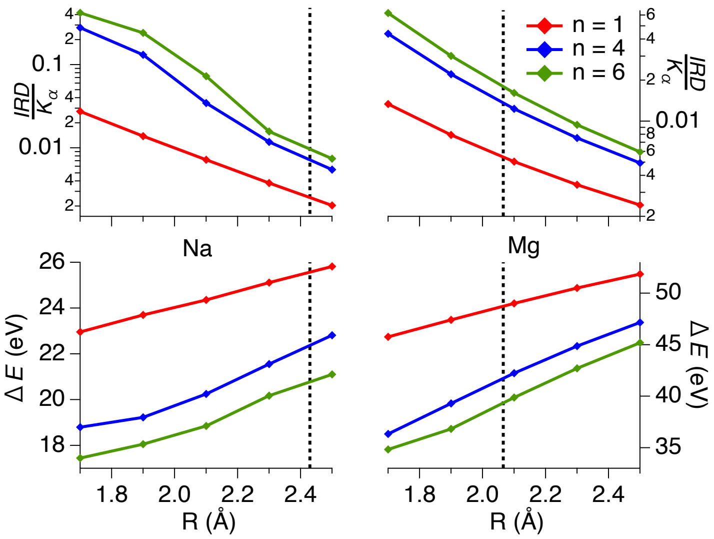

Supplementary Figure 8: Calculated IRD/K_α intensity ratio and IRD-K_α energy splitting ΔE as a function of M-water distance R and number of water molecules n for $Na^+[H_2O]_n$ (left) and $Mg^{2+}[H_2O]_n$ (right). The vertical dashed lines correspond to the experimentally determined M–O distances in solution, 2.43 Å and 2.07 Å for Na and Mg, respectively [6].

intensity, whereas for Mg, *3a*$_1$ has higher intensity than *1b*$_1$. As we will show, these differences can be understood as being caused by the different local geometries of the first solvation shell around Na and Mg.

The idealized orientation of the water molecules in the solvation shell is with the negatively charged oxygen pointing towards the M cation and the M–O direction located in the molecular plane, i.e., with the hydrogen atoms pointing away from the cation. In solution, dynamic disorder will cause the molecular orientation to differ from the idealized one.

Data from our MD simulations are shown in Supplementary Figure 9 that illustrates the dynamic disorder in the solvation shell around Na^+ and Mg^{2+} ions by showing time-traces of distance and orientation. For Na^+ and Mg^{2+}, the time-averaged distance from MD is 2.5±0.1 Å, and 2.0±0.06 Å, respectively, in quite good agreement with the experimentally determined distances of 2.43 Å and 2.07 Å, respectively [6]).

We consider two simplified types of disorder, tilting and rotation. The tilt is the angle α between the dipole vector of the individual water molecules and the radial ion–oxygen vector, see Supplementary Figure 10. In all cases, the M–O distance was kept fixed at the average value provided by MD simulations, 2.5 and 2.0 Å for Na and Mg, respectively. The idealized orientation corresponds to a tilt angle of 0°. For Na^+ and Mg^{2+}, the tilt angle from MD is 38±24°, and 15±12°, respectively, see Supplementary Figure 9. Note that ±24° for Na and ±12° for Mg is not an uncertainty of the MD simulations, but a measure of the orientational disorder in the first solvation shell.

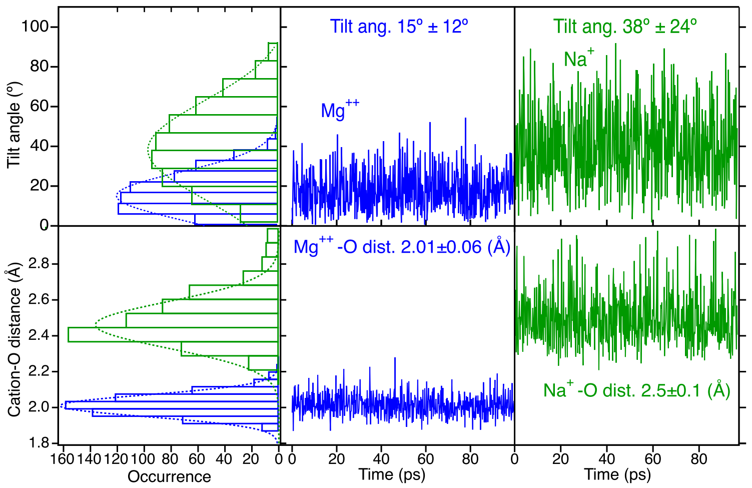

Supplementary Figure 9: The dynamic disorder of the solvation shell water molecules around Na^+ (green) and Mg^{2+} (blue) obtained from the MD simulations. The lower row shows the M–O distance, the upper row the tilt angle. Examples of time-traces from the MD simulation are shown in the middle and right columns. From the time traces, distributions of distances and tilt angles were obtained, see the left column. The distribution of distance and tilt angle values are shown as histograms, to which Gaussian fits were made. The given values for the distance and tilt angle are time averages $\pm\sigma$ from the Gaussian fits.

To explore the effects of disorder on the spectra, we have calculated IRD spectra for $Na^+[H_2O]_6$ and $Mg^{2+}[H_2O]_6$ in the HF approximation. IRD spectra as function of tilt are shown in Supplementary Figure 10.

Starting with the case of ideal orientation, $\alpha = 0°$, the Na and Mg IRD spectra are simpler in the sense that they contain fewer components. This is due to the higher symmetry of the case of ideal orientation as compared to the other cases. This warrants identifying the different spectral regions with holes in the $1b_1$, $3a_1$, and $1b_2$ molecular orbitals. The meaning of this assignment is that the cluster orbitals can be seen as mainly linear combinations of specific orbitals of the individual water molecules. We must however emphasize again that in the, more realistic, situation of a reduced or approximate symmetry for the geometry of the solvation shell, this type of labelling loses its meaning. At $\alpha = 0°$, for both Na and Mg, $3a_1$ dominates strongly, while $1b_1$ has no appreciable intensity. The $1b_2$ peak is weak for Mg, and practically absent for Na.

Turning to the case of tilting, we see that both the Na and Mg IRD spectra consist of more energetically spread components, shifted towards higher energy. This could be described as a relative weakening of the $3a_1$ and strengthening of the $1b_1$, but this is complicated by the spectral overlap of states with main $3a_1$ and $1b_1$ character. Experimentally, the Na spectrum exhibits intensity in the $1b_1$ and $3a_1$ regions of approximately equal intensity, whereas for Mg the spectrum is dominated by $3a_1$, with successively less intensity in the $1b_1$ and $1b_2$ regions. Comparing the calculated

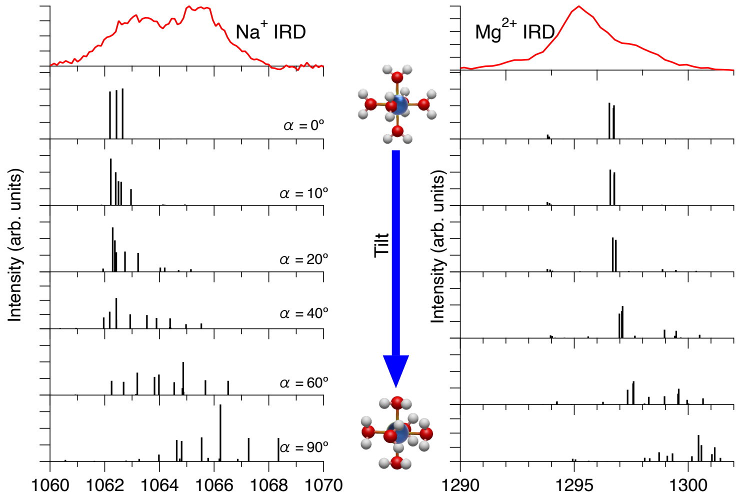

Supplementary Figure 10: Calculated IRD spectra for $Na^{+}[H_2O]_6$ and $Mg^{2+}[H_2O]_6$ with various degrees of non-ideal structure (black vertical bars), compared to the experimental spectra (red curves). Tilt refers to the angle between the ion-oxygen radial vector and the dipole vector of the individual water molecules, with 0° being the idealized structure. The same energetic shift as in Fig. 2 in the main paper has been applied to the theoretical spectra.

and experimental spectra, this indicates that the molecules on average are closer to the ideal orientation for Mg^{2+} than for Na^{+}. Our MD simulations, see Supplementary Figure 9, indicate that for $Mg^{2+}[H_2O]_6$, the tilt angle α is ~15±12°, while for $Na^{+}[H_2O]_6$ α is ~38±24°. This reflects the increase of ion–water interaction with increasing ion charge, causing the solvation shell to be more ordered around Mg^{2+} than around Na^{+}. The experimental spectra will consequently reflect a wider spread of orientations for Na^{+} than for Mg^{2+}. The calculated IRD spectra for Mg at $\alpha = 0°$, 10° and 20° agree quite well with the experimental one with a sharp dominating $3a_1$ peak. For Na at $\alpha = 40°$, the calculations predict substantial intensity in both the $3a_1$ and the $1b_1$ regions, in reasonable agreement with the experimental spectrum.

In addition to tilting, disorder can be in the form of a rotation so that one of the hydrogen atoms faces towards the ion, see Supplementary Figure 11. In the idealized orientation, the spectral shape for both Na and Mg is strongly dominated by the $3a_1$ peak. For Na, rotation makes the spectral intensity spread out in the region of the $1b_1$ peak. For Mg, the effect is similar, but less pronounced. Disorder in the form of tilt and rotation thus have similar effects in the spectral shape.

As noted in the main text, while the experimental and calculated Mg IRD spectra agree well, there is a discrepancy for Na in terms of relative intensities of $1b_1$ and$3a_1$. Experimentally $1b_1$ is somewhat stronger than $3a_1$, whereas $3a_1$ is stronger in the calculated spectra. Given the dependence of the relative intensities on the molecular orientation, this discrepancy could be due to the disorder for $Na^{+}[H_2O]_6$ being underestimated in the calculations using the representative structure.

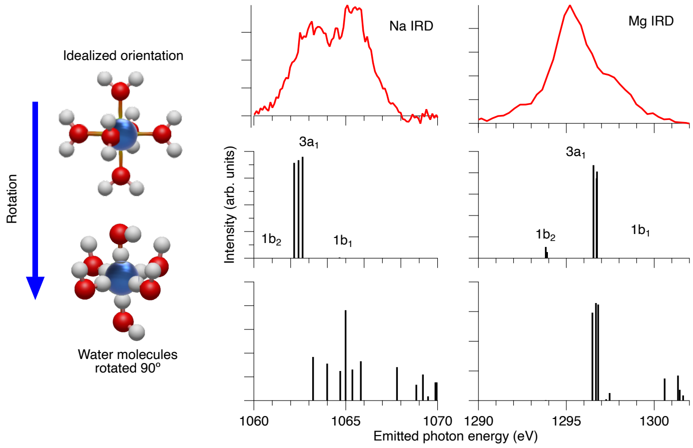

Supplementary Figure 11: (Top panels) Experimental IRD spectra compared to (middle and bottom panels) calculated IRD spectra for $Na^+[H_2O]_6$ and $Mg^{2+}[H_2O]_6$ for both the ideal rotation of the H_2O molecules where the oxygen atom points towards the metal center, as well the H_2O molecules rotated so that a hydrogen atom points towards the metal center, compare to Supplementary Figure 10. The same energetic shift as in Fig. 2 in the main paper has been applied to the theoretical spectra.

A complete simulation of the experimental spectra should include a sum of spectra calculated for a statistically representative sampling of geometries obtained as snapshots from MD simulations, which is beyond the scope of this paper. While our simulations for some selected deviations from ideal orientation are not exhaustive, they clearly demonstrate the sensitivity of IRD to the degree of orientational (dis)-order in the solvation shell.

Supplementary Note 2.7 IRD and Ion Pairing

At low concentrations, the metal cations and their Cl^- counterions are both surrounded by solvation shells consisting of only water. With increasing concentration, there may also be ion pairing in the form of a Cl^- counterion replacing a water molecule in the solvation shell of the metal cation. Based on a combination of Raman spectroscopy, MD, and quantum chemical calculations, the existence of such $Na^+ - Cl^-$ contact ion pairs with a frequency increasing with concentration has been reported for NaCl in water [7]. To investigate the effects of ion pairing on IRD, Supplementary Figure 12 compares the computed XES spectra for $M^q[H_2O]_6$ and $M^q[H_2O]_5Cl^-$ containing a contact ion pair, for $M^q = Na^+$ and Mg^{2+}.

The main predicted change is the appearance of a new band for $M^{q+}[H_2O]_5Cl^-$ at $\sim$8 eV higher energy than the IRD feature of $M^{q+}(H_2O)_6$. A simple analysis of the valence molecular orbital involved in this transition shows that it is mainly due to a small mixing of the Cl$3p$ orbital with the M$2p$ orbital. We do not observe any such transitions in the experimental spectra, indicating that the fraction of ion pairing is

low in the measured sample, in agreement with the results from Ref. [8]. The simulations, however, clearly show that IRD would be sensitive to such ion pairing. We conclude that IRD is able to probe the composition of the solvation shells, which in the investigated cases are strongly dominated by water.

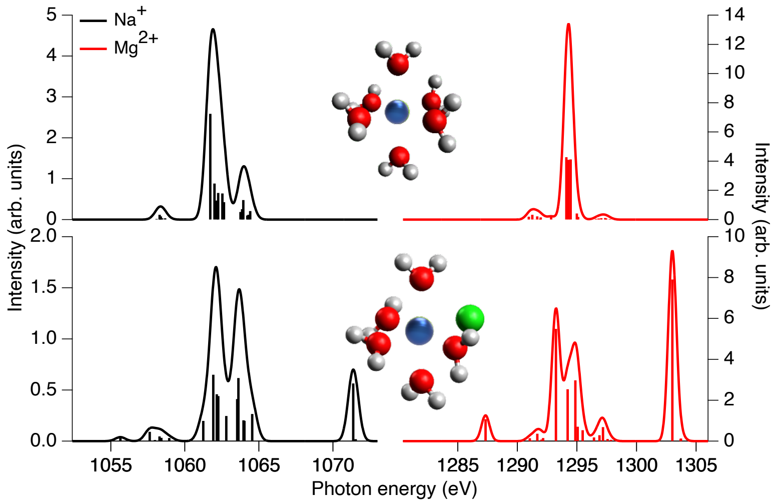

Supplementary Figure 12: XES spectra computed by Delta HF and ground-state approximation for a metal cation (blue), either Na^+ (black lines) or Mg^{2+} (red lines), surrounded by six water molecules, i.e., completely hydrated (top panel), and five water molecules and a Cl^- ion (green), i.e., a contact ion pair (bottom panel).

Supplementary References

[1] Viefhaus, J., Scholz, F., Deinert, S., Glaser, L., Ilchen, M., Seltmann, J., Walter, P., Siewert, F.: The Variable Polarization XUV Beamline P04 at PETRA III: Optics, mechanics and their performance. Nucl. Instrum. Methods Phys. Res. A **710**, 151–154 (2013)

[2] Ament, L.J.P., Veenendaal, M., Devereaux, T.P., Hill, J.P., Brink, J.: Resonant inelastic x-ray scattering studies of elementary excitations. Rev. Mod. Phys. **83**, 705–767 (2011) https://doi.org/10.1103/RevModPhys.83.705

[3] Luo, J., Wang, H., Wu, J., Romankov, V., Daffé, N., Dreiser, J.: Amyloid-beta–copper interaction studied by simultaneous nitrogen K and copper L2,3-edge soft X-ray absorption spectroscopy. iScience **24**(12), 103465 (2021) https://doi.org/10.1016/j.isci.2021.103465

[4] Manne, R.: Molecular Orbital Interpretation of X-Ray Emission Spectra: Simple Hydrocarbons and Carbon Oxides. The

Journal of Chemical Physics **52**(11), 5733–5739 (1970) https://doi.org/10.1063/1.1672852 https://pubs.aip.org/aip/jcp/article-pdf/52/11/5733/18867812/5733_1_online.pdf

[5] Jahnke, T., Hergenhahn, U., Winter, B., Dörner, R., Frühling, U., Demekhin, P.V., Gokhberg, K., Cederbaum, L.S., Ehresmann, A., Knie, A., Dreuw, A.: Interatomic and Intermolecular Coulombic Decay. Chem. Rev. **120**, 11295–11369 (2020)

[6] Persson, I.: Structure and size of complete hydration shells of metal ions and inorganic anions in aqueous solution. Dalton Trans. **53**, 15517–15538 (2024) https://doi.org/10.1039/D4DT01449A

[7] Wang, L., Morita, A., North, N.M., Baumler, S.M., Springfield, E.W., Allen, H.C.: Identification of Ion Pairs in Aqueous NaCl and KCl Solutions in Combination with Raman Spectroscopy, Molecular Dynamics, and Quantum Chemical Calculations. The Journal of Physical Chemistry B **127**, 1618–1627 (2023) https://doi.org/10.1021/acs.jpcb.2c07923

[8] Gopakumar, G., Muchová, E., Unger, I., Malerz, S., Trinter, F., Öhrwall, G., Lipparini, F., Mennucci, B., Céolin, D., Caleman, C., Wilkinson, I., Winter, B., Slavíček, P., Hergenhahn, U., Björneholm, O.: Probing aqueous ions with non-local Auger relaxation. Phys. Chem. Chem. Phys. **24**, 8661–8671 (2022)
